# *Klebsiella pneumoniae* Invasive Liver Abscess Syndrome (Klas/Ilas)—Experience of a Single Center and Up-to-Date Review of the Literature

**DOI:** 10.3390/diagnostics15121533

**Published:** 2025-06-17

**Authors:** Octavian Enciu, Elena-Adelina Toma, Valentin Calu, Dumitru Cătălin Pîrîianu, Andrei Ludovic Poroșnicu, Adrian Miron, Mircea Ioan Popa

**Affiliations:** 1 Faculty of Medicine, Carol Davila University of Medicine and Pharmacy, 05047 Bucharest, Romania; octavian.enciu@umfcd.ro (O.E.); valentin.calu@umfcd.ro (V.C.); catalin.piriianu@umfcd.ro (D.C.P.); andrei.porosnicu@umfcd.ro (A.L.P.); adrian.miron@umfcd.ro (A.M.); mircea.ioan.popa@umfcd.ro (M.I.P.); 2 Surgery Department, Elias Emergency Hospital, 011461 Bucharest, Romania; 3 The “Cantacuzino” National Military Medical Institute for Research and Development, 050096 Bucharest, Romania

**Keywords:** KLAS, *Klebsiella* liver abscess, abdominal sepsis, antimicrobial resistance, invasive liver abscess

## Abstract

**Background**: *Klebsiella pneumoniae* liver abscess (KLAS) is a potentially life-threatening condition with variable outcomes. Identifying risk factors for mortality is crucial for improving patient management. We aimed to analyze factors associated with in-hospital mortality in a cohort of patients with KLAS and review current diagnostic and treatment challenges. **Methods**: We retrospectively analyzed clinical, laboratory, microbiological, and treatment data from 20 patients admitted with KLAS. Patients were divided into survivor (*n* = 15) and non-survivor (*n* = 5) groups. Univariate analyses were performed using appropriate statistical tests to compare groups and identify mortality-related factors. **Results**: The overall in-hospital mortality rate was 25.0% (5/20). Factors significantly associated with mortality included undergoing laparotomy drainage (60.0% vs. 6.7%, *p* = 0.018) and developing in-hospital complications (80.0% vs. 6.7%, *p* = 0.002). Laparoscopic drainage was significantly associated with survival (93.3% vs. 40.0%, *p* = 0.026). Trends toward increased mortality were observed with diabetes mellitus and higher glucose levels at admission. Despite *p*-values < 0.05 from prior Fisher’s exact test, and the fact that ESBL positivity (OR = 22, 95% CI 0.86–571.32) and septic shock at admission (OR = 16.08, 95% CI 0.75–343.64) showed a very high point estimate for risk of mortality, the association was not statistically significant in our study. **Conclusions**: Mortality in this KLAS cohort was considerable. The necessity for open drainage and the development of in-hospital complications emerged as significant predictors of death, while other independent risk factor such as diabetes mellitus, high blood glucose levels at admission, septic shock at admission, and ESBL-positive strains indicated a trend towards unfavorable outcomes. These findings underscore the importance of aggressive sepsis management and addressing antimicrobial resistance. Conflicting results regarding the statistical significance of independent risk factors due to a limited sample size highlight the need for larger studies to confirm these findings.

## 1. Introduction

*Klebsiella pneumoniae* is a member of the *Klebsiella* genus in the family of Gram-negative Enterobacteriaceae that is usually found in the normal oral cavity flora and digestive system. It is also frequently identified in community-acquired infections in patients presenting with pneumonia or urinary tract infections. It is also one of the most frequent pathogens involved in hospital-acquired infections, with drug-resistant and multidrug-resistant strains being some of the biggest current threats to public health [1].

Although antimicrobial resistance is a global issue affecting patient care and increasing the burden of disease, hyperviscous, hypervirulent strains of community-acquired *K. pneumoniae* have emerged in the past decades, causing invasive *Klebsiella pneumoniae* liver abscess syndrome (KLAS/ILAS), an increasingly recognized and potentially life-threatening condition [2,3,4]. It is characterized by a primary, monomicrobial liver abscess frequently accompanied by bacteremia and subsequent metastatic spread to the eyes, soft tissue, and central nervous system, causing endophthalmitis, necrotizing fasciitis, and meningitis in the absence of concurrent hepatobiliary disease or other sites of primary *K. pneumoniae* infection [5]. Spreading of the disease is most likely through fecal–oral transmission, but whether the pathway is through the bloodstream and ulterior preferential seeding in the liver or bacterial translocation from the digestive tract to the liver and subsequent bacteremia and metastasis to distant sites is still a matter of research [6,7]. Risk factors for the development of KLAS are Asian heritage, diabetes mellitus, obesity, old age, and alcoholism, but it can also appear in otherwise healthy individuals.

Initially described predominantly in Southeast Asia, particularly Taiwan, KLAS is now being reported increasingly frequently in other regions, including Europe [8]. The incidence and prevalence are very heterogenous among countries and even continents nowadays, with more than 60% of *K. pneumoniae* infections being with hvKp in Asia, while countries such as Belgium, Italy, Germany, and the Netherlands reported over 10% hvKp in a May 2025 study [9]. Moreover, *K. pneumoniae* is mentioned increasingly often across continents as a main pathogen involved in liver abscesses, alongside *E. coli*, *Streptococcus* spp., *Enterococcus* spp., and *Entamoeba histolytica* [10]. Romania presents a unique epidemiological landscape due to factors such as increasing antibiotic resistance, healthcare-associated infections, and potential underdiagnosis of hypervirulent *K. pneumoniae* strains. However, data on KLAS’s prevalence, clinical presentation, and microbiological characteristics in this region remain limited. While standard *Klebsiella* liver abscesses may remain localized to the liver, KLAS can lead to severe complications and even death if not promptly diagnosed and treated. Therefore, its emergence poses a diagnostic and therapeutic challenge for physicians unfamiliar with this distinct clinical entity.

This review aims to provide a comprehensive analysis of updates in KLAS diagnosis and treatment strategies in Europe, with a specific focus on Romania, while presenting the experience of a single center in diagnosing and treating this syndrome. By synthesizing the available literature, epidemiological data, and clinical case reports, we seek to highlight the burden of KLAS, its microbiological features, risk factors, and treatment challenges. Furthermore, we discuss the implications for diagnosis, antimicrobial stewardship, and public health strategies to mitigate the spread of this emerging syndrome.

## 2. Materials and Methods

We retrospectively reviewed the data regarding patients admitted in the General Surgery Department of the Elias Emergency University Hospital between October 2020 and December 2024 to identify patients diagnosed with liver abscesses. Furthermore, we analyzed microbiological data from blood and abscess cultures and included all patients with positive results for *K. pneumoniae*. The VITEK 2 system (bioMérieux, Marcy l’Etoile, France) and BD Phoenix (Becton Dickinson, Oxford, UK) were used to confirm bacterial identification among the available isolates. To determine the capsular types of *K. pneumoniae*, *cps* genotyping by the polymerase chain reaction (PCR) detection of K serotype-specific alleles, including K1 and K2, was performed, using GoTaq^®^ G2 Flexi DNA Polymerase (Promega, Fitchburg, WI, USA) [11]. Demographical data, comorbidities, risk factors, metastatic sites, treatment strategies, and outcomes were recorded. Patients with incomplete data, those with concurrent COVID-19 infections, and those with polymicrobial infections or monomicrobial infections caused by other pathogens (*Escherichia coli* and *Enterococcus* spp.) were excluded from the study.

Statistical analysis was carried out using IBM SPSS Statistics 25. Continuous variables were compared using independent samples *t*-tests (assuming unequal variances where appropriate) or Mann–Whitney *U* tests. Categorical variables were compared using Fisher’s exact test. A *p*-value < 0.05 was considered statistically significant. To further quantify the strength of association between categorical baseline factors and in-hospital mortality, odds ratios (ORs) and their 95% confidence intervals (CIs) were calculated.

We also searched multiple databases (PubMed, Google Scholar, Science Direct, and Web of Science) for updates in the diagnosis, treatment, and microbiological profile of *K. pneumoniae* causing pyogenic liver abscesses.

## 3. Results

Out of 57 patients diagnosed with liver abscesses during their admission in our department, 24 had positive single-pathogen *K. pneumoniae* blood and/or liver abscess cultures. Records were incomplete for four of the patients, most likely because they were admitted during the height of the SARS-CoV-2 pandemic. They were subsequently excluded from the study. The demographic data, the presence of risk factors, laboratory test results, metastatic sites, antimicrobial profile, drainage procedures employed, and outcomes are presented in Table 1.

The mean age was 70.1 ± 10.6 years, and 11 (55.0%) patients were male. Common comorbidities included diabetes mellitus (*n* = 13, 65.0%) and obesity (*n* = 11, 55.0%). No patients in this cohort had underlying cirrhosis or malignancy.

Most abscesses were located exclusively in the right lobe of the liver (*n* = 16, 80.0%). All patients had positive abscess cultures, and 14 (70.0%) had positive blood cultures. Two patients (10.0%) had ESBL-producing organisms isolated. One phenotypic analysis was performed in our cohort, and the K1 capsular gene was identified. At admission, the mean temperature was 38.2 ± 0.6 °C, the mean white blood cell count was 19.9 ± 6.4 × 10^3^/µL, and the median procalcitonin (PCT) was 12.7 ng/mL (IQR 6.4–>100).

Septic shock was diagnosed at admission in 11 (55.0%) patients, and 3 patients (15%) had septic metastases (2 of them presented with endophthalmitis, and 1 patient had cutaneous metastases). The most common drainage procedure performed was laparoscopic drainage of the abscess (*n* = 16, 80.0%), including four patients who initially underwent unsuccessful percutaneous drainage and subsequently underwent laparoscopic drainage; the remaining patients underwent laparotomy and open drainage (*n* = 4, 20.0%). All patients received broad-spectrum intravenous antibiotics since admission: those who were diagnosed with septic shock received meropenem, while the others received either ceftriaxone and metronidazole or piperacillin/tazobactam and metronidazole. These were adjusted according to their evolution and reevaluation, and de-escalation or escalation of antibiotherapy was performed according to susceptibility profiles after the antibiogram results. All patients that were discharged followed prolonged oral antibiotherapy for up to 4 weeks (ciprofloxacine or levofloxacine) and were reevaluated with ultrasound at 2 weeks and CT scans at 4 weeks after discharge.

Five patients (25%) presented complications during hospitalization, with three of them developing MODS, one patient was diagnosed with a stroke, and one patient suffered thrombosis of the right hepatic vein.

### Mortality Analysis

The overall in-hospital mortality rate was 25.0% (5/20 patients). Table 2 details a comparison of characteristics between survivors (*n* = 15) and non-survivors (*n* = 5).

Several factors at admission were significantly associated with increased mortality. Non-survivors were significantly more likely to present with septic shock (100.0% vs. 40.0%, *p* = 0.026) and to have ESBL-positive isolates (40.0% vs. 0.0%, *p* = 0.026) than survivors. However, although ESBL positivity (OR = 22.14) and septic shock at admission (OR = 16.08) exhibited large point estimates for increased odds of mortality, their respective 95% CIs (0.86–571.32 for ESBL; 0.75–343.64 for septic shock) included 1.0. This indicates that, when assessed by the CI of the OR, these associations did not achieve statistical significance in this cohort, despite *p*-values < 0.05 from prior Fisher’s exact tests.

Furthermore, non-survivors were significantly more likely to undergo laparotomy (60.0% vs. 6.7%, *p* = 0.018) and significantly less likely to undergo laparoscopy (40.0% vs. 93.3%, *p* = 0.026) as the drainage procedure. The occurrence of in-hospital complications was significantly higher among non-survivors (80.0% vs. 6.7%, *p* = 0.002). The development of complications was also associated with significantly increased odds of mortality (OR = 56.00; 95% CI: 2.83–1109.49), as was undergoing open drainage (OR = 21.00; 95% CI: 1.40–314.6). Conversely, laparoscopic drainage was associated with significantly lower odds of mortality (OR = 0.048; 95% CI: 0.00–0.71).

Trends toward higher mortality were observed in patients with diabetes mellitus (100.0% vs. 53.3%, *p* = 0.071), positive blood cultures (100.0% vs. 60.0%, *p* = 0.107), and recent antibiotic use prior to admission (80.0% vs. 33.3%, *p* = 0.089). Most of these factors also had 95% CIs that included 1.0, with positive blood cultures (OR = 14.05; 95% CI: 0.35–160.87), diabetes mellitus (OR = 9.71; 95% CI: 0.46–206.4), and recent antibiotic use (OR = 8.00; 95% CI: 0.70–91.80) indicating trends rather than statistical significance. The considerable width of many of these CIs highlights the limited precision of the estimates due to the small sample size.

**Figure 1:**
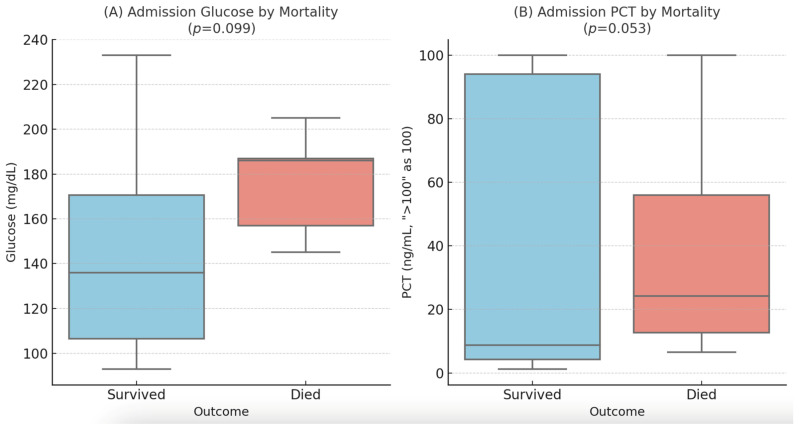
Distribution of admission glucose (**A**) and procalcitonin (**B**) levels by in-hospital mortality. Box plots display the median (line), interquartile range (box), and 1.5 × IQR (whiskers). PCT values > 100 ng/mL were treated as 100 ng/mL for visualization.

Higher admission glucose levels also showed a trend towards association with mortality (mean 176.0 vs. 145.5 mg/dL, *p* = 0.099). Median admission PCT levels were higher in non-survivors (24.2 ng/mL) compared to survivors (8.7 ng/mL), but this difference did not reach statistical significance (*p* = 0.053). These findings are illustrated in Figure 1.

There were no statistically significant differences between survivors and non-survivors in terms of age, sex, obesity, liver lobe involvement (right only vs. bilateral), admission temperature, WBC count, platelet count, AST, ALT, BUN, CRP levels, or length of hospital stay.

## 4. Discussion

This case series provides insights into the clinical presentation, management, and outcomes of 20 patients with *Klebsiella pneumoniae* liver abscess (KLAS) in a European hospital setting. Our findings highlight the significant morbidity associated with this condition, characterized by a high prevalence of comorbidities, severe initial presentation, and notable mortality. KLAS, primarily caused by hypervirulent *Klebsiella pneumoniae* (hvKp), has emerged as a significant health concern beyond its initial identification in East Asia, with increasing reports in Europe, including Romania. The right lobe of the liver is affected more frequently than the left lobe and the caudate lobe, probably due to the portal venous drainage of the mesenteric veins, which is unequally distributed, with the inferior mesenteric vein draining preferentially in segments 5–8 [12,13]. We also observed this imbalance in our cohort, with 80% of patients only displaying radiologically identifiable disease in the right lobe. The clinical management of KLAS is complicated by the pathogen’s evolving antimicrobial resistance (AMR), necessitating a multifaceted approach encompassing accurate diagnosis, adaptative antimicrobial stewardship, and adequate public health strategies.

### 4.1. Diagnosis and Risk Factors

Accurate and timely diagnosis of KLAS is critical for effective treatment and prevention of complications. However, the non-specific clinical presentation of KLAS, often resembling other hepatic or systemic infections, poses diagnostic challenges. Advanced imaging techniques, such as contrast-enhanced computed tomography (CT) or magnetic resonance imaging (MRI), are instrumental in identifying liver abscesses. Definitive diagnosis requires microbiological confirmation through culture and sensitivity testing of aspirated abscess material or blood cultures.

KLAS has been associated with several risk factors that predispose individuals to infection. Diabetes mellitus (DM) is consistently identified as a significant risk factor, with studies indicating that up to 63% of patients with KLAS have DM [14]. Diabetes mellitus was highly prevalent in our cohort (65.0%), and although DM only showed a trend towards significance for mortality in our univariate analysis (*p* = 0.071), its high prevalence suggests it may be an important underlying factor contributing to the overall morbidity in this patient population, consistent with the literature identifying DM as a key risk factor for KLAS. Poor glycemic control in diabetic patients may impair neutrophil phagocytosis, increasing susceptibility to infection. However, a study by Chuang et al. published in 2016 that evaluated 230 patients with KLAS found that 35–57.9% of patients had DM, and clinical outcomes were not different between them and non-diabetics. Moreover, the six virulent capsular types (K1, K2, K5, K20, K54, and K57) were more prevalent in the patients who did not have DM and those who exhibited good glycemic control in the DM group [15].

Recent antibiotic use has also been implicated as a predisposing factor for KLAS. Antibiotic administration within 30 days prior to infection has been associated with an increased risk of developing KLAS [14]. In animal models, ampicillin, vancomycin, or azithromycin administration predisposed *K. pneumoniae*-colonized mice to increased liver abscess formation [16]. We did not find any significant correlation in our cohort regarding this issue.

Geographical and ethnic factors appear to influence the prevalence of KLAS. The syndrome has been reported predominantly in Asian countries, including Taiwan and Korea, and even in case series reported in the Americas, 40–60% of patients were of Asian origin or had traveled to Asian countries recently [4,17]. Studies have yet to identify any host genetic factors that can explain such a high prevalence among Asians. Genetic predisposition for bacterial translocation, as well as a high prevalence of intestinal carriage of hypervirulent K1 and K2 strains compared to that reported for other ethnicities, might explain this imbalance, but a certain incriminating mechanism has eluded science so far [4,7,14].

Other identified risk factors for KLAS include male sex, advanced age, liver cirrhosis, and malignancy. A recent study focusing on the prognostic risk factors for pyogenic liver abscess caused by *K. pneumoniae* found that these conditions may increase susceptibility to infection [2].

Understanding these risk factors is crucial for the early identification and management of individuals at increased risk for KLAS, particularly in regions like Romania, where the syndrome may be underrecognized. However, despite a slight male predominance (55% of patients), other factors often associated with KLAS mortality in larger studies, such as age, malignancy, or cirrhosis (absent in this cohort), or certain laboratory markers, did not show statistical significance here, possibly due to the limited sample size. There was a strong association between septic shock at admission and mortality (*p* = 0.026) in our study, consistent with findings from the literature, identifying sepsis and shock as major independent risk factors for death in KLAS patients. This statistical significance, nevertheless, based on Fisher’s exact test *p*-values, had 95% confidence intervals for its respective odds ratio that included 1.0, warranting cautious interpretation of this specific association in this cohort.

Metastatic spread is one of the main characteristics of KLAS, and it is often associated with higher rates of morbidity and mortality than non-metastatic disease and other types of pyogenic liver abscesses. Risk factors for septic metastases include septic shock at admission, low platelet count, low albumin, high levels of C-reactive protein (over 200 mg/L), and abscesses larger than 6 cm, as reported in a case-controlled study in Singapore [18]. Patients with KLAS often develop endophthalmitis, uveitis, chorioretinitis, or retinal abscesses, underscoring the importance of early recognition and management to prevent permanent visual impairment [19]. Meningitis is another serious complication associated with KLAS. The hypervirulent strains of *Klebsiella pneumoniae* responsible for KLAS have a heightened ability to invade the central nervous system, leading to meningitis [20]. Necrotizing fasciitis, a rapidly progressing soft tissue infection, has also been reported in association with KLAS [21]. Additionally, KLAS can lead to other metastatic infections, such as septic pulmonary emboli and abscesses in the lungs, adrenal glands, prostate, and psoas muscles, and can also rarely determine osteomyelitis or even endocarditis [22]. In our cohort, one out of three patients (33.3%) with metastatic sites at admission died—patients with endophthalmitis were in the survivor group but suffered irreversible eyesight loss in the eye affected by metastatic spread. However, the presence of metastatic sites was not statistically associated with mortality in this study (*p* > 0.99). While metastatic spread often indicates severe disease, the lack of statistical significance here is likely due to the very small number of patients with metastatic complications in this dataset.

The overall mortality rate in this cohort was 25.0%, which appears notably higher than rates typically reported in recent European studies, often cited between 6% and 19% [23,24]. This discrepancy may reflect the small sample size or potentially a higher severity of illness within this specific patient group.

### 4.2. Treatment

The management of *Klebsiella* liver abscess syndrome (KLAS) necessitates a comprehensive approach that integrates antimicrobial therapy with procedural interventions to eradicate the infection and prevent complications. While antibiotic intravenous or even oral therapy alone can be employed in some cases, a study published in 2024 showed that this course of treatment was the most expensive, as well as the least effective, when compared to combined antibiotics and drainage or surgical resection of septic foci in the liver [25]. Sample collection for blood and abscess cultures is necessary to establish an accurate KLAS diagnosis, to appraise disease extension, and to tailor the antimicrobial therapy according to local resistance profiles. Abscess cultures should be performed even in patients who have already started empiric antibiotic treatment at admission. All the patients in our cohort underwent long-term antibiotic treatment as indicated by the Infectious Diseases consult but needed drainage procedures, which is why they were admitted to the surgical ward. Abscess samples were collected intraoperatively for all patients, and 16 of them also had blood samples collected at admission, with 13 positive cultures with *K. pneumoniae*.

#### 4.2.1. Antimicrobial Therapy and Emerging Therapeutic Developments

Empirical antibiotic treatment should be initiated promptly, targeting Gram-negative pathogens, focusing on *Klebsiella pneumoniae*. Third-generation cephalosporins, such as ceftriaxone or cefotaxime, are commonly employed as first-line agents. In regions with a high prevalence of extended-spectrum beta-lactamase (ESBL)-producing strains, carbapenems like imipenem or meropenem are preferred due to their broader spectrum of activity. The typical duration of intravenous antibiotic therapy ranges from 2 to 3 weeks, followed by an additional 2 to 4 weeks of oral antibiotics (usually ciprofloxacin or trimethoprim-sulfamethoxazole) to ensure complete eradication of the pathogen [26]. However, the total duration of treatment, whether initial intravenous antibiotic therapy is advisable, and choosing the proper moment to switch to oral treatment remain a topic of debate, heightened by the evidence of progressing AMR rates [27]. Evidence in the literature is unclear and data from randomized clinical trials are very scarce, but one such study demonstrated that early step-down to oral antibiotics was non-inferior to prolonged intravenous therapy in terms of treatment success, suggesting that oral therapy can be a viable option in patients with non-metastatic disease and without the criteria for sepsis or septic shock [28]. Patients in our cohort who did not present in septic shock and had a favorable postoperative course received third-generation cephalosporins during admission and adequate antibiotherapy at home, as outlined above. Patients admitted in septic shock and those with metastatic disease received meropenem at admission and had a longer course of intravenous antibiotherapy before switching to oral antibiotics.

The rise of multidrug-resistant *K. pneumoniae* strains has prompted the development of novel antimicrobial agents. Aztreonam/avibactam, a monobactam and a beta-lactamase inhibitor combination, has shown promise against metallo-beta-lactamase-producing Enterobacterales, including resistant *K. pneumoniae* strains. This combination was approved for medical use in the European Union in April 2024 and in the United States in February 2025, offering a new therapeutic option for infections caused by multidrug-resistant Gram-negative bacteria [29,30].

#### 4.2.2. Drainage Procedures

Drainage of the liver abscess is a critical component of treatment, in addition to antimicrobial therapy. Percutaneous drainage, guided by imaging techniques such as ultrasound or computed tomography, is preferred due to its minimally invasive nature and high success rates. This approach not only aids in reducing the bacterial load but also provides material for culture and sensitivity testing, facilitating targeted antibiotic therapy.

In cases where percutaneous drainage is not feasible or fails to achieve adequate abscess resolution, surgical intervention may be necessary. This was the case for four of our patients, and surgical drainage was necessary due to the inability to drain thick, viscous, or loculated pus adequately. In our cohort, laparoscopic drainage was the most frequently employed drainage method (80.0%). Notably, laparoscopy was significantly associated with survival (93.3% in survivors vs. 40.0% in non-survivors, *p* = 0.026), while open drainage was significantly associated with mortality (60.0% in non-survivors vs. 6.7% in survivors, *p* = 0.018). Open surgical drainage was typically reserved for patients who had contraindications for laparoscopic procedures and those with multiloculated abscesses or rupture into the peritoneal cavity. The association between laparotomy and higher mortality likely reflects the selection bias, where open surgery was reserved for more critically ill patients or those failing less invasive methods. The observed association of laparoscopy with better outcomes in this cohort aligns with its role as a safe and effective minimally invasive option, potentially preferred over open surgery when surgical drainage is indicated and feasible.

The extent of the surgical procedure varies greatly in the literature, depending on individual patient characteristics, from a simple drainage of the abscess cavity to partial liver resections [31]. Liver resections were not necessary in our cohort.

### 4.3. Microbiological Profile and Antimicrobial Stewardship

KLAS is predominantly associated with hypervirulent strains, notably those expressing capsular serotypes K1 and K2, but other capsular types that express hyperviscosity include serotypes K5, K20, K54, and K57 [15]. These serotypes are characterized by a hypermucoviscous phenotype, contributing to their increased virulence and greater expression of siderophores (yersiniabactin, salmochelin, and aerobactin) [14]. This hallmark of hypervirulent strains is associated with the presence of the magA and rmpA genes, which enhance capsule production and increase resistance to phagocytosis, as well as contributing to the pathogen’s ability to cause severe infections, including metastatic complications like endophthalmitis and meningitis [32]. Moreover, hvKp strains have demonstrated the ability to survive longer after intestinal colonization than classical strains of *K. pneumoniae* [33].

Traditional serotyping methods like the Quellung reaction rely on specific antisera to detect capsular polysaccharides. However, these methods can be labor-intensive, require specialized reagents, and may lack sensitivity and specificity [34]. Molecular techniques have been developed to overcome these limitations. Multiplex PCR assays targeting genes specific to K1 and K2 serotypes, such as the wzi gene, have been utilized for rapid and accurate identification. For instance, a study developed a single multiplex PCR assay targeting seven virulence factors and the wzi gene specific for the K1 and K2 capsular serotypes, demonstrating its utility in the surveillance of emerging hypervirulent strains [35].

The genetic diversity and complex population structure of *K. pneumoniae* complicate the analysis and interpretation of genomic data. Additionally, the presence of multiple capsular types and the potential for horizontal gene transfer can lead to misidentification. A genomic surveillance framework and genotyping tools have been introduced to address these challenges, aiming to provide a standardized approach for genomic analysis [36]. While these genotypes, particularly K1/K2 and the presence of *rmpA/rmpA2*, are strong indicators of a strain’s potential to express the HMV phenotype, the correlation is not absolute [37]. Relying solely on genotyping, as in our retrospective analysis focusing on K1/K2 serotypes, provides valuable information about the potential hypervirulence of the isolates but is an indirect assessment and may not fully capture the phenotypic expression of hypermucoviscosity. A comprehensive understanding of an isolate’s virulence potential in KLAS would ideally involve both the genotypic characterization of key virulence and capsular loci and phenotypic assessment of hypermucoviscosity while keeping in mind that not all strains that cause pyogenic liver abscesses display this phenotype [33,38]. Advancements in rapid diagnostic techniques, such as matrix-assisted laser desorption/ionization time-of-flight (MALDI-TOF) mass spectrometry and polymerase chain reaction (PCR)-based assays, have enhanced the ability to quickly identify *K. pneumoniae* and its resistance profiles. These tools facilitate the timely initiation of appropriate antimicrobial therapy, potentially improving patient outcomes.

The increasing AMR in *K. pneumoniae* strains complicates the therapeutic landscape of KLAS. Studies from Romanian healthcare settings have reported high resistance rates in *K. pneumoniae*, particularly to third-generation cephalosporins and carbapenems. For instance, a study conducted at the “Victor Babes” Hospital of Infectious Diseases and Pneumology in Timisoara in 2023 found that *K. pneumoniae* exhibited significant resistance rates, with 41.41% in the first quarter of the year [39]. Another study from a tertiary hospital in Bucharest reported increased carbapenem resistance from 32.3% in 2019 to 54.5% in 2021 [40].

A critical finding in our study was the association between ESBL positivity and mortality (*p* = 0.026), with both ESBL-positive patients succumbing to their illness. While only two cases were identified, and that could explain the lack of significance regarding the odds of mortality as stated beforehand, this finding aligns with the growing global concern regarding antimicrobial resistance in pathogens causing KLAS. In Europe, while Streptococcus spp. and E. coli are often cited as common causative organisms, *Klebsiella pneumoniae* (often implicated in ESBL production) is an emerging challenge [1,41]. ESBL complicates treatment, potentially contributing to the observed poor outcomes, and underscores the importance of rapid microbiological diagnosis and susceptibility testing.

In 2014, Popescu et al. reported the first documented case of KLAS in Romania, which identified the K1 capsular serotype as the causative agent [42]. Subsequently, in 2018, Paraschiv et al. described a severe case involving a female patient who exhibited meningeal, ocular, and cutaneous metastatic infections [43]. In the one case in our study where the samples were phenotypically analyzed, the K1 serotype was also identified, and the patient had a favorable outcome. We did not find any articles nationwide reporting the more aggressive K2 serotype, any of the other rarer serotypes, or gene expressions associated with hvKp.

### 4.4. Public Health Strategies for Control and Prevention

Table 3 summarizes key characteristics and outcomes from selected recent European studies focusing on KLAS. This comparative overview includes details on study populations, reported mortality rates, hypervirulent strains detected, and identified risk factors, allowing for an assessment of regional trends and variations concerning this syndrome. Moreover, this summary shows that, as was the case in our study, it is no longer common for patients with KLAS to be of Asian heritage or to have traveled to Asian countries in the last year. The number of cases reported in most of these European studies is still significantly lower than those reported in Asian countries, but both ESBL strains and K1 or K2 serotypes have been reported [44].

Addressing the emergence and spread of KLAS requires comprehensive public health interventions. Emerging therapeutic strategies, including phage therapy, biofilm-disrupting agents, and monoclonal antibodies, are under investigation and hold promise for future prevention and treatment modalities [45]. One preclinical study published in 2019, as well as a comprehensive review published in 2024 regarding the development of a vaccine against *K. pneumoniae*, outline that there are encouraging results, especially with the aid of artificial intelligence and advanced computational models for manufacturing effective immunotherapies [46,47]. Until such a time, enhanced surveillance systems are essential to monitor the incidence and resistance patterns of *K. pneumoniae* infections. Infection control measures, including strict adherence to hygiene protocols and isolation procedures in healthcare settings, are vital to prevent nosocomial transmission [48]. Public health education campaigns can raise awareness among healthcare professionals and the public about the risks associated with KLAS and the importance of early diagnosis and appropriate treatment [49]. Given the documented cases and rising AMR rates in Romania, national health authorities should prioritize KLAS in their infectious disease agendas, promoting research, surveillance, and the development of targeted interventions.

**Table 3:** Summary of selected European studies on KLAS with reported hypervirulent strains, metastatic sites, and mortality rates.

Country	Author(s)	Year	Study Design	No. of Patients	No. of Patients with KLAS	Patients of Asian Heritage	Metastatic Sites (No of Patients)	ESBL Rate	K1/K2 Strain	Key Risk Factors for Mortality/Severity	Mortality Rate
Ireland	Moore et al. [50]	2013	Case report	3	3	2	NA *	NA	K1 (1), K2 (2)	NA	NA
Norway	Kirstoffer Holmas [51]	2014	Case report	3	3	1	NA	NA	K1 (3)	NA	0%
Spain	Cubero et al. [52]	2015	Prospective, single-centre	878 isolates	14 isolates	NA	NA	NA	K1 (16), K2 (3)	DM *, malignancy, renal failure, hvKP *	9 pts with hvKP
France	Rafat et al. [53]	2018	Prospective, single-centre	59	2	NA	NA	NA	K1 (5), K2 (5)	hvKP strains	50% (all patients with hvKP, not just KLAS)
Spain	Ruiz-Hernandez et al. [54]	2019	Retrospective, single-centre	193	19	NA	NA	3%	NA	DM	11%
Spain	Sanchez-Lopez et al. [55]	2019	Case report	4	4	0	ocular (2)	NA	K1 (2), K2 (2)	NA	0%
France	Martellosio et al. [56]	2022	Retrospective, single centre	33	8	0	ocular, lung, meningeal, kidney (8)	NA	K1 (5), K2 (2)	Metastases, hvKP strains, immunosuppressive drugs	12.5%
Sweden	Svensson et al. [57]	2023	Retrospective, observational	364	24	NA	NA	4%	NA	NA	NA

* NA = data not available; DM = diabetes mellitus; hvKp = hypervirulent *Klebsiella pneumoniae.*

## 5. Conclusions

In conclusion, this analysis highlights the significant mortality risk associated with KLAS, particularly in patients who develop complications during hospitalization, as well as those who warrant laparotomy for abscess drainage, within this specific cohort. While limited by sample size, the findings emphasize the need for prompt diagnosis, aggressive management of sepsis, appropriate antimicrobial therapy guided by susceptibility testing to combat resistance, and careful selection of drainage procedures based on patient stability and abscess characteristics. This is the first series of cases describing KLAS patients in Romania, and further larger, prospective studies, ideally multicenter European collaborations, are needed to delineate the risk factors better and optimize a robust antimicrobial stewardship, as well as integrate comprehensive public health strategies, to mitigate the impact of this syndrome.

## 6. Study Limitations

The small cohort size (*n* = 20) is the most significant limitation. This restricts the statistical power to detect true differences between groups and increases the risk of chance findings. Comparisons involving very small subgroups lack robustness. Non-parametric tests were used to mitigate the presence of threshold values, but these markers’ exact magnitude of difference is uncertain. Due to the limited sample size and number of events, performing multivariable logistic regression to identify independent risk factors is not statistically sound and was therefore not conducted. Furthermore, the small number of mortality events limited the statistical power for identifying risk factors and resulted in wide 95% confidence intervals for many odds ratio estimates, reducing their precision. Consequently, some factors (such as ESBL positivity and septic shock at admission) that showed statistical significance based on Fisher’s exact test *p*-values had 95% confidence intervals for their respective odds ratios that included 1.0, warranting cautious interpretation of these specific associations. Ultimately, while the string test is a valuable assay for phenotypic characterization, it was not routinely used per the protocol in our clinical microbiology laboratory for the patients retrospectively analyzed in this cohort.

## Figures and Tables

**Table 1:** Baseline characteristics, clinical features, and laboratory findings of the study cohort.

Cohort Features	Overall Data (*n* = 20)
*Demographics*	
Age (years), mean ± SD	70.1 ± 10.6
Male Sex, *n* (%)	11 (55.0%)
*Comorbidities*	
Obesity, *n* (%)	11 (55.0%)
Diabetes Mellitus, *n* (%)	13 (65.0%)
Cirrhosis, *n* (%)	0 (0.0%)
Malignancy, *n* (%)	0 (0.0%)
*Clinical Presentation*	
Right Lobe Only, *n* (%)	16 (80.0%)
Right + Left Lobe, *n* (%)	4 (20.0%)
Metastatic Sites, *n* (%)	3 (15.0%)
Positive Abscess Culture, *n* (%)	20 (100.0%)
Positive Blood Culture, *n* (%)	14 (70.0%)
ESBL-Positive, *n* (%)	2 (10.0%)
Recent ATB Use (<30 d), *n* (%)	9 (45.0%)
Septic Shock at Admission, *n* (%)	11 (55.0%)
Temperature at Admission (°C), mean ± SD	38.2 ± 0.6
*Laboratory Findings (at Admission)*	
WBC (×10^3^/µL), mean ± SD	19.9 ± 6.4
Platelets (×10^3^/µL), mean ± SD	263 ± 186
AST (U/L), mean ± SD	105 ± 70
ALT (U/L), mean ± SD	90 ± 69
BUN (mg/dL), mean ± SD	66.9 ± 35.2
Glucose (mg/dL), mean ± SD	152.9 ± 42.3
CRP ^1^ (mg/L), median (IQR)	263 (199–363)
PCT ^1^ (ng/mL), median (IQR)	12.7 (6.4->100)
*Intervention + Outcomes*	
Laparoscopic Drainage, *n* (%)	16 (80.0%)
Open Drainage, *n* (%)	4 (20.0%)
Length of Stay (days), mean ± SD	12.7 ± 5.3
Complications, *n* (%)	5 (25.0%)
Mortality, *n* (%)	5 (25.0%)

^1^ CRP values >450 mg/L and PCT values >100 ng/mL were treated as censored data and considered as the highest values reported by the laboratory.

**Table 2:** Comparison of the characteristics between survivors and non-survivors.

	Survived (*n* = 15)	Died (*n* = 5)	Odds Ratio (95% CI)	*p*-Value *
Demographics				
Age (years), mean ± SD	68.3 ± 10.1	75.6 ± 11.4	-	0.227 ^1^
Male Sex, *n* (%)	8 (53.3%)	3 (60.0%)	1.31(0.17–10.26)	>0.999 ^2^
Comorbidities				
Obesity, *n* (%)	8 (53.3%)	3 (60.0%)	1.31 (0.17–10.26)	>0.999 ^2^
Diabetes Mellitus, *n* (%)	8 (53.3%)	5 (100.0%)	9.71 ^4^ (0.46–206.40)	**0.071 ^2^**
Clinical Presentation				
Right Lobe Only, *n* (%)	13 (86.7%)	3 (60.0%)	0.23 (0.02–2.37)	0.241 ^2^
Right + Left Lobe, *n* (%)	2 (13.3%)	2 (40.0%)	4.33 (0.42–44.43)	0.241 ^2^
Metastatic Sites, *n* (%)	2 (13.3%)	1 (20.0%)	1.63 (0.11–22.98)	>0.999 ^2^
Positive Blood Culture, *n* (%)	9 (60.0%)	5 (100.0%)	14.05 (0.35–160.87)	**0.100 ^2^**
ESBL-Positive, *n* (%)	0 (0.0%)	2 (40.0%)	22.14 ^4^ (0.86–571.32)	**0.026 ^2^**
Recent ATB Use (<30 d), *n* (%)	5 (33.3%)	4 (80.0%)	8.00 (0.70–91.80)	**0.089 ^2^**
Septic Shock at Admission, *n* (%)	6 (40.0%)	5 (100.0%)	16.08 ^4^ (0.75–343.64)	**0.026 ^2^**
Temperature at Admission (°C), mean ± SD	38.1 ± 0.6	38.4 ± 0.7	-	0.308 ^1^
Laboratory Findings (at admission)				
WBC (×10^3^/µL), mean ± SD	19.2 ± 6.8	22.1 ± 5.0	-	0.313 ^1^
Platelets (×10^3^/µL), mean ± SD	263 ± 199	263 ± 161	-	0.996 ^1^
AST (U/L), mean ± SD	96 ± 72	130 ± 66	-	0.378 ^1^
ALT (U/L), mean ± SD	84 ± 73	108 ± 62	-	0.506 ^1^
BUN (mg/dL), mean ± SD	61.1 ± 37.7	84.0 ± 23.1	-	0.208 ^1^
Glucose (mg/dL), mean ± SD	145.5 ± 42.5	176.0 ± 25.9	-	**0.099 ^1^**
CRP (mg/L), median (IQR)	239 (170–323)	344 (165–>450)	-	0.249 ^3^
PCT (ng/mL), median (IQR)	8.7 (1.5–56.0)	24.2 (6.5–>100)	-	**0.053 ^3^**
*Intervention + Outcomes*				
Laparoscopic Drainage ^1^, *n* (%)	14 (93.3%)	2 (40.0%)	0.048 (0.00–0.71)	**0.026 ^2^**
Open Drainage, *n* (%)	1 (6.7%)	3 (60.0%)	21.00 (1.40–314.06)	**0.018 ^2^**
Length of Stay (days), mean ± SD	13.1 ± 5.3	11.4 ± 5.8	N/A	0.588 ^1^
Complications, *n* (%)	1 (6.7%)	4 (80.0%)	44.33 ^4^ (2.83–1109.49)	**0.002 ^2^**

^1^ Independent samples *t*-test (assuming unequal variances where appropriate). ^2^ Fisher’s exact test. ^3^ Mann–Whitney *U* test. Note: PCT > 100 treated as 100 for calculations; CRP > 450 treated as 450 for calculations. ^4^ OR calculated using Haldane–Anscombe correction (0.5 added to all cells) due to presence of zero in one cell of the 2 × 2 contingency table. * Bold *p*-values indicate statistical significance (*p* < 0.05) or near-significance (*p* < 0.10).

## Data Availability

The raw data supporting the conclusions of this article will be made available by the authors on request.

## Abbreviations

The following abbreviations are used in this manuscript:

AMR	Antimicrobial Resistance
AST	Aspartate Aminotransferase
ALT	Alanine Aminotransferase
ATB	Antibiotic
BUN	Blood Urea Nitrogen
CI	Confidence Interval
CRP	C-Reactive Protein
CT	Computed Tomography
DM	Diabetes Mellitus
ESBL	Extended-Spectrum Beta-Lactamase
hvKp	Hypervirulent *Klebsiella pneumoniae*
ILAS	Invasive Liver Abscess Syndrome
IQR	Interquartile Range
K1/K2	Capsular Serotypes K1/K2 (of *K. pneumoniae*)
KLAS	*Klebsiella pneumoniae* Liver Abscess Syndrome
LOS	Length of Stay
MALDI-TOF	Matrix-Assisted Laser Desorption/Ionization Time-of-Flight
MDR	Multidrug Resistance
MODS	Multiple-Organ Dysfunction Syndrome
MRI	Magnetic Resonance Imaging
OR	Odds Ratio
PCR	Polymerase Chain Reaction
PCT	Procalcitonin
SD	Standard Deviation
T	Temperature (° Celsius)
WBC	White Blood Cell count
